# Multi-Scale CNN-LSTM for Short-Circuit Fault Diagnosis of Shipboard Power System

**DOI:** 10.3390/s26092754

**Published:** 2026-04-29

**Authors:** Xun Chen, Kaikai You, Xiaoqiang Dai

**Affiliations:** College of Automation, Jiangsu University of Science and Technology, Zhenjiang 212100, China; justlab@163.com (X.C.); ykk_mail@163.com (K.Y.)

**Keywords:** shipboard power system, short-circuit fault diagnosis, multi-scale CNN-LSTM, Shapley value

## Abstract

Shipboard power systems are essential to the safe and stable operation of marine vessels, while short-circuit faults may lead to equipment damage and system interruption under complex onboard operating conditions. To improve fault diagnosis performance in this setting, this study proposes an interpretable short-circuit fault diagnosis framework that combines a multi-scale CNN-LSTM model with Shapley value analysis. Relative changes between pre-fault and fault-state electrical signals are used to construct the input representation, which helps characterize fault-related variations more effectively. The multi-scale convolution branches extract patterns associated with different temporal ranges, and the LSTM layer further models their sequential dependence. Shapley value analysis is introduced to quantify the contribution of voltage- and current-related features, identify the most informative inputs, and support feature screening. Experiments on a Simulink-based shipboard power system dataset show that the proposed method achieves competitive fault diagnosis performance compared with baseline models, including CNN, LSTM, and LightGRU. Under repeated runs, the proposed framework attains an average diagnostic accuracy of 99.03 ± 0.20%, while also maintaining strong precision, recall, and F1-score performance. Under the tested noise conditions, it shows better robustness than the comparison methods. These results indicate that the proposed framework can provide accurate and interpretable fault diagnosis for shipboard power systems.

## 1. Introduction

Shipboard power systems (SPS) form the critical backbone of modern marine vessels, ensuring the reliable operation of navigation, propulsion, and communication modules. However, the compact architecture and dynamic operational environments of SPS make them particularly susceptible to short-circuit faults, which necessitate robust protection strategies [1,2,3]. Comprehensive reviews of SPS and microgrid architectures have repeatedly highlighted the urgent need for rapid and accurate fault diagnosis to prevent cascading system failures under highly variable working conditions [4,5].

To address these diagnostic challenges, researchers have extensively explored data-driven and machine learning applications in power system protection [6,7]. Early intelligent methodologies predominantly relied on traditional algorithms, such as Support Vector Machines (SVM) for adaptive fault identification [8], neuro-fuzzy inference systems [9], and deep neural networks combined with discrete wavelet transforms (DWT) [10]. Furthermore, spectral analysis tools, including advanced Fast Fourier Transform (FFT) techniques, have been widely used to extract frequency-domain features from transient signals [11,12,13]. While these conventional approaches laid the groundwork for automated fault detection, they generally struggle with the complex, non-stationary feature extraction required for shipboard dynamics.

Driven by the need for higher diagnostic accuracy, deep learning architectures have recently dominated the field of power system diagnostics. Advanced frameworks, including CNN-based transformers [14,15] and hybrid machine learning models [16], have shown immense potential in enhancing grid resilience. To effectively capture temporal dependencies in electrical signals, researchers have increasingly integrated Convolutional Neural Networks (CNN) with Gated Recurrent Units (GRU) [17] and Long Short-Term Memory (LSTM) networks [18]. Similarly, innovative wavelet-based CNNs and Graph Neural Networks (GNNs) have been introduced for non-intrusive monitoring and fault detection in marine environments [19,20], while standalone LSTM models remain popular for large-scale transmission line classification [21]. Despite their superior performance under steady-state conditions, these single-scale deep learning models often lack the sensitivity required to distinguish subtle transients from normal operational variations.

To overcome the limitations of single-scale feature extraction, multi-scale convolutional structures have been heavily investigated across various industrial applications [22]. By fusing multi-scale CNNs with BiLSTM or BiGRU, these architectures have demonstrated exceptional efficacy in identifying complex fault signatures in rotating machinery and wind turbines under varying noise conditions [23,24,25,26,27]. Concurrently, to better visualize and understand the high-dimensional feature spaces generated by these complex models, neighbor embedding methods such as t-SNE and UMAP have become essential analytical tools [28,29,30]. However, despite their high accuracy and improved feature representations, the inherent “black-box” nature of these deep learning models remains a significant hurdle.

In safety-critical applications like SPS, industrial operators require transparent decision-making processes. Consequently, Explainable Artificial Intelligence (XAI) techniques have recently emerged to bridge the gap between high performance and interpretability in energy systems [31,32]. Groundbreaking approaches utilizing XAI-driven frameworks have been successfully applied to DC microgrids [33] and decentralized smart grids [34] to ensure trustworthy fault location and prediction. Furthermore, recent hybrid models combining ensemble learning with XAI have demonstrated significant potential in identifying faults within complex renewable energy systems, such as wind turbines [35]. Nevertheless, a comprehensive framework that integrates multi-scale temporal modeling with feature-level interpretability (such as SHAP) specifically tailored for shipboard power systems is still lacking.

To clearly illustrate the research gaps and position our proposed framework within the current academic landscape, Table 1 provides a comparative summary of recent state-of-the-art fault diagnosis methodologies.

The main contributions of this work are summarized as follows:

(1) A relative-change-based feature construction strategy is developed for short-circuit fault diagnosis in shipboard power systems. By comparing pre-fault and post-fault electrical characteristics and incorporating the fundamental-frequency amplitude, the proposed input representation improves fault sensitivity while reducing dependence on specific operating conditions.

(2) A multi-scale CNN-LSTM diagnosis model is proposed to capture fault-related patterns at different temporal scales. By integrating parallel convolutional branches and temporal sequence modeling, the model improves the discrimination of fault categories with similar transient characteristics.

(3) A SHAP-based feature interpretation and validation mechanism is introduced to quantify the contribution of electrical features, verify their faithfulness through deletion retention tests, and guide feature screening for more reliable and interpretable diagnosis.

## 2. Problem Formulation and Data Preparation

### 2.1. Short-Circuit Fault Conditions in Shipboard Power Systems

Shipboard power systems operate in a compact and strongly coupled electrical environment in which generators, converters, cables, and loads interact continuously. When a short-circuit fault occurs, voltage and current waveforms change abruptly and then evolve under the combined effects of system dynamics, protection devices, and control actions. Therefore, short-circuit fault diagnosis in shipboard power systems can be naturally formulated as a multi-class pattern recognition problem with strong transient and temporal characteristics.

In this study, the diagnosis task is defined as a five-class supervised classification problem. The five operating conditions are NORMAL, LG, LL, LLG, and LLL, where LG denotes a single-line-to-ground fault, LL denotes a line-to-line fault, LLG denotes a double-line-to-ground fault, and LLL denotes a three-phase fault. The objective of the proposed framework is to assign each waveform sample to one of these five operating categories according to its waveform-derived feature representation.

The diagnosis framework directly learns discriminative patterns from electrical measurement signals rather than relying on explicit analytical fault equations. Each sample contains a pre-fault interval and a post-fault interval, and the model performs fault-type recognition based on compact descriptors extracted from the corresponding voltage and current waveforms.

### 2.2. Signal Acquisition and Data Description

**Figure 1:**
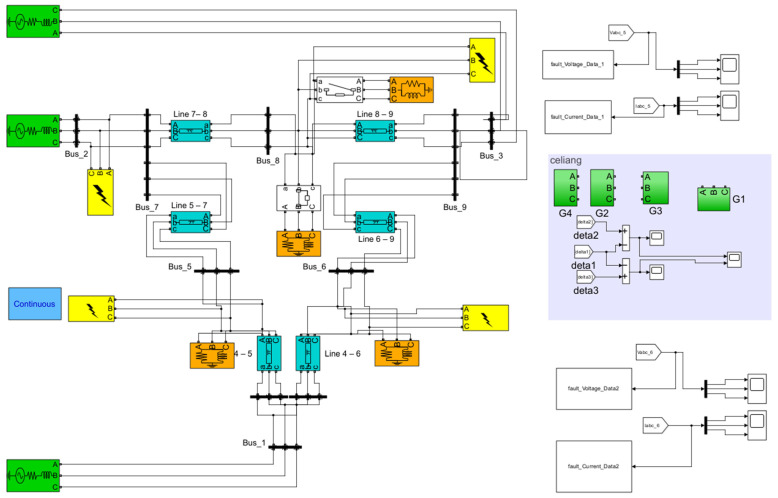
Simulation model for shipboard power system.

The dataset used in this study was generated from a shipboard power system simulation model developed in Simulink, as shown in Figure 1. The simulated system is centered on three synchronous generators and adopts a ring-connected distribution architecture. It integrates multiple buses, transmission lines, and load units, thereby enabling the simulation of both normal operating conditions and representative short-circuit events in a controlled and repeatable manner.

A total of 1200 labeled samples were generated and distributed across five operating conditions: NORMAL (88 samples), LG (324 samples), LL (293 samples), LLG (297 samples), and LLL (198 samples). For the fault cases, different faulted phase combinations were considered for LG, LL, and LLG conditions. In addition, the fault initial phase angle was set to 0°, 45°, or 90° and the short-circuit resistance was set to 1 Ω, 5 Ω, or 10 Ω. Faults were applied at four different branches of the simulated shipboard network, as illustrated in Figure 1.

For each sample, the recorded signals consist of three-phase voltage and three-phase current waveforms, namely *V_a_*, *V_b_*, *V_c_*, *I_a_*, *I_b_*, and *I_c_*. These waveform records were exported as individual sample files and labeled according to the corresponding simulated operating condition. During preprocessing, the complete dataset was divided by stratified sampling into training, validation, and test subsets to preserve class proportions across subsets. According to the implemented preprocessing pipeline, the final split ratio is 60% for training, 20% for validation, and 20% for testing.

### 2.3. Event-Aligned Segmentation Strategy

In order to analyze the relative changes caused by faults, an event-aligned segmentation strategy is used to divide each waveform into pre-fault and post-fault segments. This method approximates the moment of fault occurrence by a fixed proportion of the time period, ensuring consistency in the processing. The goal is to provide a common reference for subsequent feature design through this segmentation method so that each sample is processed in a consistent manner.

**Figure 2:**
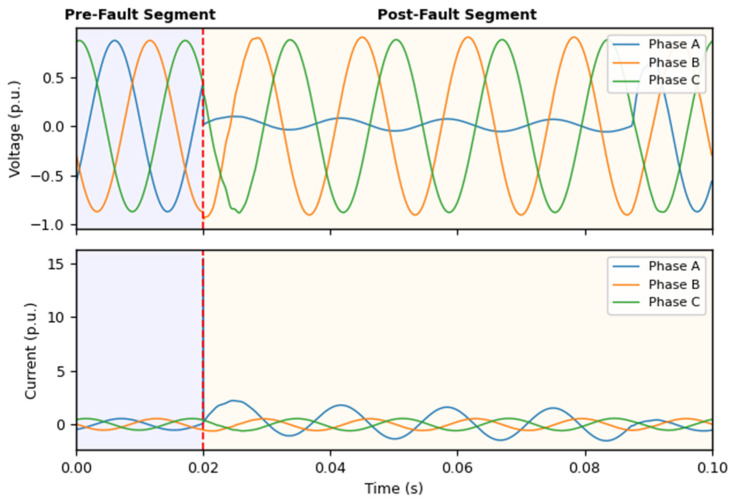
Event-aligned segmentation of a short-circuit waveform.

To explicitly demonstrate this process, Figure 2 illustrates the actual three-phase voltage and current waveforms of a representative single-line-to-ground (LG) fault on Phase A. As indicated by the red dashed line at t = 0.02 s, the pre-fault segment captures the steady-state operation, while the post-fault segment records the immediate physical response. The actual input signals clearly show a severe voltage sag and a prominent current surge in the faulted phase (Phase A), alongside subsequent dynamic variations in the healthy phases. By separating these two stages based on precise event timing, the feature construction process can accurately isolate the fault-induced changes from the normal operating baseline. This treatment effectively reduces the influence of absolute operating levels and emphasizes the contrast behavior that is most relevant for diagnostic decision-making.

### 2.4. Feature Construction

Based on the segmented three-phase voltage and current waveforms, a compact feature set was constructed from *V_a_*, *V_b_*, *V_c_*, *I_a_*, *I_b_*, and *I_c_*.

(1) Time-Domain Contrast Features

The time-domain features were designed to characterize both the operating-state contrast and the disturbance intensity caused by short-circuit faults. First, the RMS values of three-phase voltages and currents were calculated from the pre-fault and fault-state intervals, respectively. These descriptors retain information from both stages and allow the model to learn the electrical differences between normal and faulted conditions.

To further describe the severity of post-fault transients, several fault-state indicators were extracted from the current and voltage signals. Specifically, the peak values of three-phase voltages and currents were included to reflect the maximum disturbance amplitude. In addition, the crest factor of each phase current was calculated to describe waveform sharpness under fault conditions, and the current energy in the fault-state interval was used to measure the cumulative intensity of the disturbance. Together, these features provide a compact description of both steady-to-fault variation and post-fault transient behavior.

(2) Fundamental Amplitude Characteristics

To complement the time-domain representation, frequency-domain features were extracted from the fault-state interval. Instead of relying on full-spectrum modeling, this study focused on the amplitude of the fundamental-frequency component of three-phase voltages and currents. This choice was made because the fundamental component remains closely related to the system electrical state, whereas higher-frequency components are more susceptible to switching effects, signal non-stationarity, and measurement noise.

In addition, an approximate total harmonic distortion (THD) indicator was calculated for each phase current to provide a coarse description of harmonic distortion after fault occurrence. As a result, the initial handcrafted feature pool contains 33 candidate features, including RMS descriptors, peak descriptors, current crest-factor features, current energy features, fundamental-amplitude features, and current-THD features. These features form the basis for subsequent feature screening and model input construction.

### 2.5. Feature Selection and Normalization

After feature extraction, the candidate feature set was further refined through feature selection and normalization before being fed into the diagnosis model. To retain the most discriminative variables while reducing redundancy, Analysis of Variance (ANOVA) was adopted as a univariate feature-ranking method, and the top 15 features were selected using the SelectKBest strategy. The retained features were drawn from both time-domain and frequency-domain descriptors and covered voltage- and current-related characteristics.

To ensure a fair evaluation, feature selection was performed after dataset splitting and was fitted only on the training set. The same transformation was then applied to the validation and test sets. After feature selection, standard scaling was used to normalize the retained features so that each feature had zero mean and unit variance. The scaler was likewise fitted on the training set and then applied to the validation and test sets. This preprocessing workflow avoids information leakage and improves the stability of model training.

The final selected features were arranged as a one-dimensional feature sequence and used as the input to the proposed multi-scale CNN-LSTM model. This compact representation preserves the most relevant diagnostic information while reducing redundancy in the original feature pool.

## 3. The Proposed Multi-Scale CNN-LSTM Fault Diagnosis Framework

### 3.1. Overall Framework

**Figure 3:**
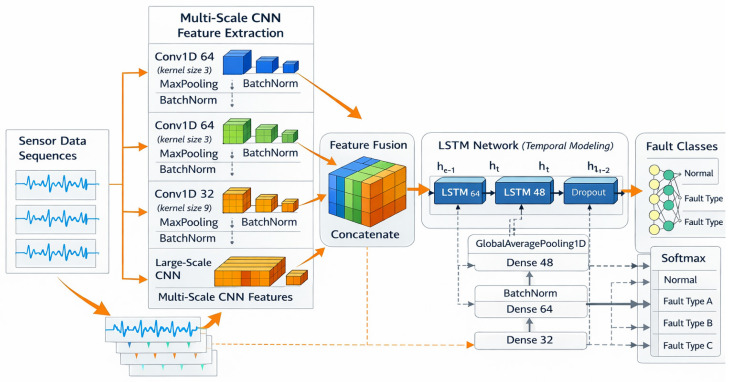
Overall framework of the multi-scale CNN-LSTM model.

The overall framework of the proposed fault diagnosis method for shipboard power systems, together with the architecture of the multi-scale CNN-LSTM model, is shown in Figure 3. The framework combines compact feature-based input representation, multi-scale convolutional feature extraction, temporal dependency modeling, and final fault classification. Starting from the electrical measurement signals of shipboard power systems, the method learns discriminative fault characteristics from constructed voltage- and current-related features and outputs the predicted fault category.

**Figure 4:**
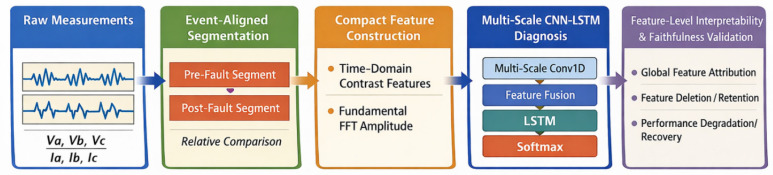
Overall framework of the proposed feature-level interpretable fault diagnosis method.

The model input, illustrated in Figure 4, is an ordered feature sequence constructed from multi-channel voltage and current measurements after preprocessing. A multi-scale convolutional feature extraction module is first employed to learn fault-related patterns under different receptive fields. Specifically, multiple parallel one-dimensional convolutional branches with different kernel sizes are used to capture feature interactions at different scales. This design enables the model to represent both fine-grained local variations and broader feature dependencies contained in the diagnostic input. Similar multi-scale strategies have been applied in shipboard power grid fault diagnosis and related time-series analysis tasks [36,37].

The feature maps extracted by different branches are then fused through concatenation and further processed by an LSTM module. The role of the LSTM is to model the dependency structure within the ordered feature representation and enhance the integration of information learned from different convolutional branches. Since short-circuit fault characteristics are reflected through coupled changes in multiple electrical descriptors, such dependency modeling is beneficial for improving diagnostic discrimination. Related recurrent modeling approaches have also shown good performance in fault diagnosis and condition monitoring [38,39].

Finally, the learned representation is passed through fully connected layers, and a SoftMax layer is used to output the probabilities of different fault categories. Through the combination of multi-scale feature extraction and sequence dependency modeling, the proposed framework provides a structured and effective solution for short-circuit fault diagnosis in shipboard power systems.

### 3.2. Input Representation

After feature construction and selection, each sample is represented as a one-dimensional sequence of 15 selected features. These retained features are drawn from the initial handcrafted feature pool and include both time-domain and frequency-domain descriptors, covering voltage- and current-related characteristics extracted from the pre-fault and fault-state intervals. In this way, the input preserves compact but diagnostically relevant electrical information for short-circuit fault identification.

To facilitate subsequent convolutional and sequence modeling, the selected features are arranged in a fixed order and represented in tensor form. This representation enables the network to process features with different physical meanings within a unified input structure and to learn correlations among them. Rather than directly operating on raw waveforms, the model takes the ordered feature sequence as input, which provides a more compact representation for diagnosis.

This input design can improve training stability and enhance tolerance to noise and redundant information. Compared with raw waveform input, the use of compact feature sequences reduces input dimensionality while retaining essential fault-related information. In addition, by jointly incorporating voltage- and current-related descriptors, the adopted representation helps improve the robustness and data efficiency of shipboard fault diagnosis. The purpose of this design is not to replace end-to-end learning but to provide a practical balance among compactness, interpretability, and diagnostic performance.

### 3.3. Multi-Scale Convolutional Feature Extraction

To characterize fault features at different scales, a multi-scale one-dimensional convolutional feature extraction module is designed. This module consists of multiple parallel convolutional branches with different kernel sizes, which learn local feature patterns under different receptive fields. Through this parallel structure, the model can capture complementary fault-related information from the ordered input feature sequence.

Let the input feature sequence be denoted as *X*. The output of the *i*-th convolutional branch is given by Equation (1).

(1) $$F_{i}=Conv_{ki}\left(X\right),i=1,2,\ldots ,N$$

where $k_{i}$ denotes the kernel size and $F_{i}$ represents the extracted feature map. Since different branches use different kernel sizes, each branch focuses on a different range of feature interactions. Smaller kernels are more sensitive to fine-grained local variations, whereas larger kernels are able to capture broader dependency patterns in the input representation.

The outputs of all branches are connected together along the feature dimension, and the fused feature is expressed as Equation (2).

(2) $$Z=\left[F1,F2,\ldots ,FN\right]$$

where Z the fused feature representation. This multi-scale fusion strategy enables the model to preserve complementary information learned by different convolutional branches and provides a richer representation for subsequent fault diagnosis.

$X\in R^{T\times d}$ denotes the input feature sequence, where T is the sequence length and *d* is the feature dimension. For the adopted compact input representation, T corresponds to the number of ordered selected features, *k_i_* is the kernel size of the *i*-th convolutional branch, *F_i_* represents the extracted feature map, and [.] denotes concatenation along the feature channel dimension.

### 3.4. Temporal Modeling with LSTM

After multi-scale feature fusion, an LSTM network is employed to model dependencies in the fused feature sequence. Through its gating mechanism, the LSTM selectively retains and updates informative content across sequential steps, which makes it suitable for further integrating the complementary information extracted by the multi-scale convolutional branches.

Let the fused feature sequence be denoted as $\left\{Z_{i}\right\}$.The LSTM updates its hidden state at each time step as Equation (3).

(3) $$h_{t}=LSTM\left(z_{t,},h_{t-1}\right)$$

$Z_{i}$ denotes the fused feature vector at time step t; $h_{t}$ and denote the current and $h_{t-1}$ previous hidden states, respectively. By recursively updating the hidden state, the LSTM captures the dependency structure within the fused feature sequence and enhances the representation of fault-related patterns learned from different convolutional scales.

The hidden state at the final step is used as the temporal representation for subsequent classification since it summarizes the sequentially aggregated fault-related information in the fused feature space [38,39].

### 3.5. Fault Classification and Training Configuration

After time modeling, the final feature representation is input into the fully connected layer, followed by the SoftMax classifier to generate the failure category probability, which is expressed as Equation (4).

(4) $$\overset{\wedge }{y}=soft\max(Wh+b)$$

The model is trained by minimizing the cross-entropy loss function, which is expressed as Equation (5).

(5) $$l=-\sum_{c=1}^{C}y_{c}\log\left(\overset{\wedge }{y}\right)$$

W and b are trainable parameters; C denotes the number of fault classes; y and $\overset{\wedge }{y}$ denote the ground-truth label vector and the predicted probability vector, respectively.

The main architectural and training configurations of the proposed model are summarized in Table 2 to ensure reproducibility. Feature visualization techniques are further employed to analyze the learned representations and support interpretation of the diagnostic results.

## 4. Experimental Results and Analysis

To verify the effectiveness of the proposed fault diagnosis framework, we conducted four types of experiments on the constructed dataset:

1. Performance comparison: Compare the proposed framework with baseline models to evaluate its advantages in fault diagnosis tasks.

2. Ablation study: Analyze the role of key components in the framework through ablation experiments and explore the impact of each module on diagnostic performance.

3. Robustness evaluation: Conduct experiments under different noise levels to assess the model’s robustness and stability in variable environments.

4. Convergence and feature analysis: Analyze the model’s convergence and feature behavior to verify its reliability in practical applications.

These experiments comprehensively evaluate the performance of the framework and reveal its performance and potential under different operating conditions.

### 4.1. Performance Comparison

To ensure the statistical significance and reliability of the results, all models were independently trained and evaluated 5 times with different random seeds. The results in Table 3 and the subsequent ablation studies are presented as mean ± standard deviation. Table 3 presents the proposed multi-scale CNN-LSTM model that exhibits superior performance, achieving an average accuracy of 99.03 + 0.20% and an F1-score of 99.03 ± 0.19% after multiple independent runs. While the standard deviation is relatively higher than other models, this variability reflects the model’s robustness across different noise levels and fault conditions. The model’s ability to consistently achieve high accuracy, even with varying noise interference (up to 10 dB), confirms its effectiveness for fault diagnosis in shipboard power systems under real-world conditions. This indicates that a framework combining multi-scale spatial feature extraction and temporal feature modeling can effectively improve the accuracy and robustness of short-circuit fault diagnosis in shipboard power systems. The repeated-run results provide a more reliable estimate of model performance and reduce the uncertainty associated with single-run evaluation.

The experimental results validate the advantages of the multi-scale CNN-LSTM model, particularly in handling complex fault signals, as it is better at capturing spatiotemporal features and surpasses traditional single-structure deep learning models and lightweight gated recurrent models. This demonstrates the effectiveness of the multi-scale CNN-LSTM model in fault diagnosis of shipboard power systems, providing strong support for practical applications.

**Figure 5:**
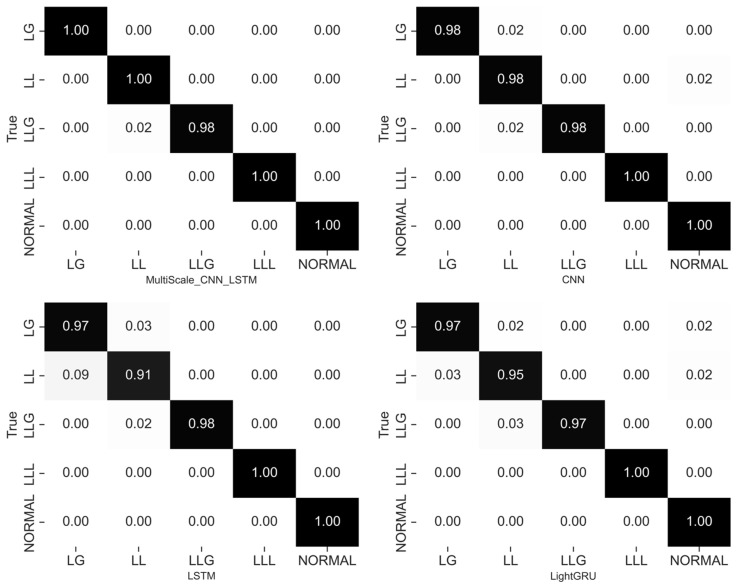
Confusion matrices of different diagnostic models.

The confusion matrix results in Figure 5 reveal a strong diagonal dominance for the proposed multi-scale CNN-LSTM model. This characteristic indicates the model’s high classification accuracy across all fault categories. In contrast, the benchmark models (CNN, LSTM, and LightGRU) have higher misclassification rates for fault types with similar transient characteristics. Specifically, the multi-scale CNN-LSTM model can more accurately distinguish different fault modes. Its classification performance is particularly prominent for fault categories with pronounced transient features such as short-circuit faults.

The reason for this phenomenon is that short-circuit fault waveforms usually have similar transient characteristics. Although traditional CNN, LSTM, and LightGRU models can extract spatial features, they are prone to misclassification when dealing with these similar features. In contrast, the multi-scale CNN-LSTM model can effectively capture dynamic information at different scales by extracting features from multiple scales, enhancing the ability to distinguish these similar fault types. Moreover, the LSTM module further enhances the model’s ability to model temporal dependencies and dynamic features, enabling accurate capture of the evolution process of fault responses.

The experimental results indicate that the proposed multi-scale CNN-LSTM approach can achieve balanced and reliable fault classification, effectively reducing misclassification, thereby providing stronger discriminative ability in multi-class fault diagnosis. Notably, while maintaining high diagnostic accuracy, the proposed method also demonstrates a relatively small standard deviation, indicating the stability and robustness of the multi-scale CNN-LSTM framework in handling stochastic variations during training.

### 4.2. Ablation Study

**Figure 6:**
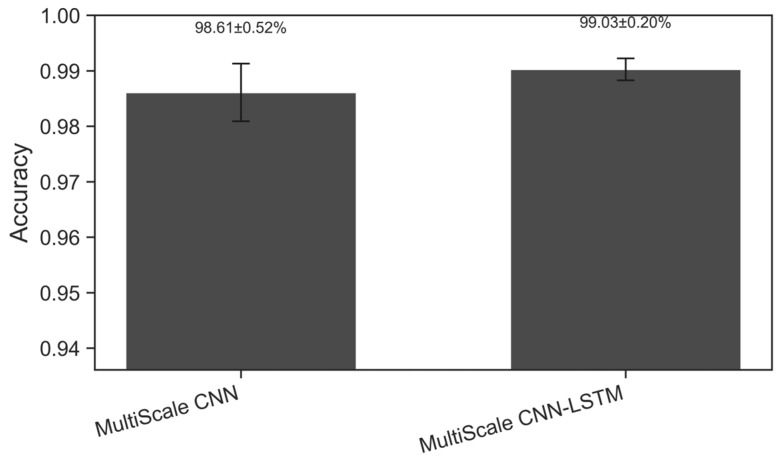
Ablation test: diagnostic accuracy of multi-CNN and multi-CNN-LSTM.

The results in Figure 6 show a significant improvement in diagnostic accuracy for the multi-scale CNN-LSTM model with the LSTM module. This performance is compared to that of the standalone multi-scale CNN model. The multi-scale CNN model can extract static spatial features from fault signals and identify some fault patterns. However, short-circuit faults typically have distinct transient and temporal variations. These variations cannot be captured solely through spatial features. By modeling the temporal dependencies of the signal, the LSTM module effectively captures the dynamic evolution of short-circuit fault responses. This further enhances the diagnostic accuracy of the model.

Experimental results indicate that a framework combining multi-scale spatial feature extraction with LSTM temporal modeling can more comprehensively characterize the spatiotemporal characteristics of fault signals, enhancing the robustness and accuracy of diagnosis. The experimental results validate the critical role of temporal modeling in improving diagnostic performance and further demonstrate the necessity of integrating the LSTM module into the multi-scale CNN model.

**Figure 7:**
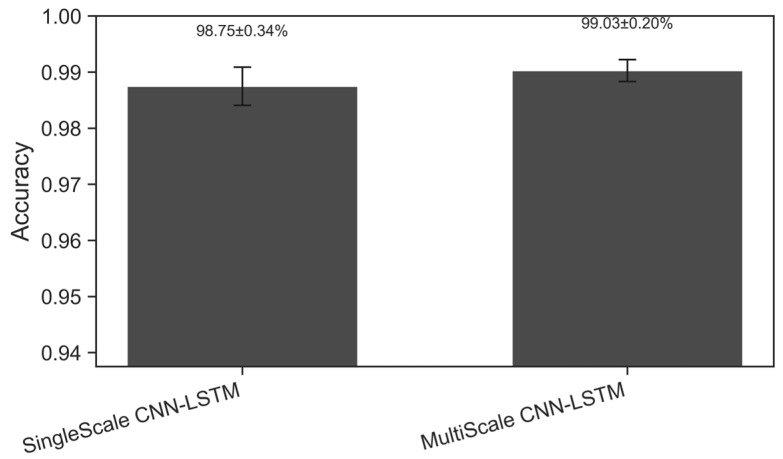
Ablation test: diagnostic accuracy of single-scale-CNN-LSTM and multi-scale-CNN-LSTM.

The results in Figure 7 show that the CNN-LSTM model with multi-scale convolution significantly outperforms the single-scale convolution version in terms of accuracy. A single-scale convolution model can extract certain fault features. However, its limited receptive field prevents it from fully capturing all dynamic features in short-circuit fault signals. In contrast, multi-scale convolution is able to extract features at multiple scales. It effectively captures the diverse temporal characteristics of short-circuit fault waveforms in shipboard power systems. This design thus improves the model’s diagnostic accuracy.

The results of this ablation experiment indicate that multi-scale convolutional feature extraction is crucial for improving the performance of shipboard power system fault diagnosis. By integrating multi-scale spatial features, the model can more comprehensively capture the complex temporal characteristics of short-circuit faults, further enhancing diagnostic accuracy.

### 4.3. Robustness Evaluation Under Noise Interference

**Figure 8:**
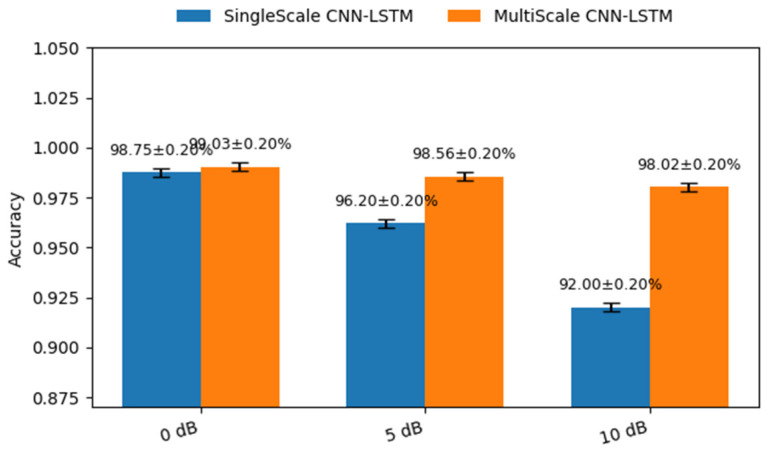
Diagnostic accuracy under different noise levels.

The results in Figure 8 show the performance of the proposed multi-scale CNN-LSTM model is superior to that of the single-scale CNN-LSTM model, even under noise-free conditions. As noise increases and SNR decreases, the performance gap between the two models becomes more pronounced. At SNRs of 0 dB and 5 dB, the multi-scale CNN-LSTM model maintains stable performance. Meanwhile, the accuracy of the single-scale model drops significantly. When the SNR further decreases to 10 dB, the accuracy of all models declines. Even so, the multi-scale CNN-LSTM model still retains a clear advantage.

The noise experiment results indicate that the proposed multi-scale model can effectively extract fault-related features and effectively suppress noise interference, demonstrating strong robustness and adaptability, suitable for application in actual shipboard power systems.

### 4.4. Convergence and Feature Analysis

**Figure 9:**
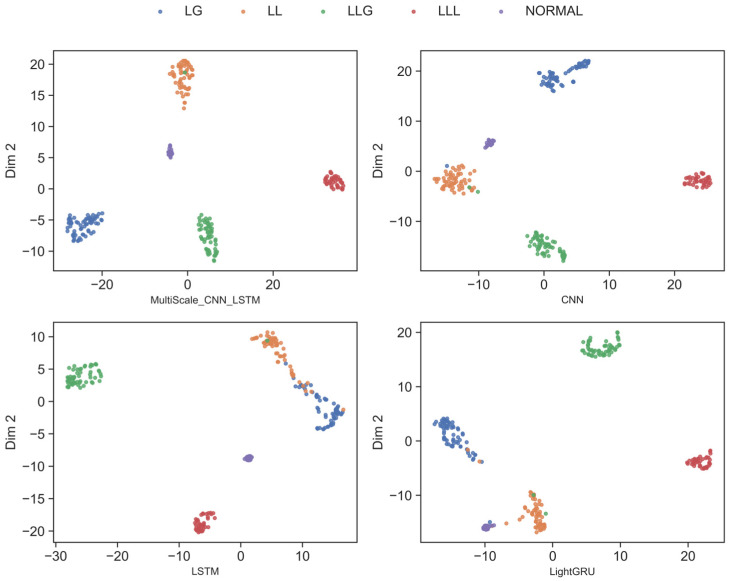
Visualization of feature extraction in two dimensions.

The results in Figure 9 show the two-dimensional feature distribution learned by the model. In this figure, features for each fault type and the normal state form tight clusters in space. There is a clear separation between different fault categories. For example, there is almost no overlap between normal operating conditions and fault types. Similar faults (such as LG, LL, and LLG) cluster together, showing their similarity in the feature space.

**Figure 10:**
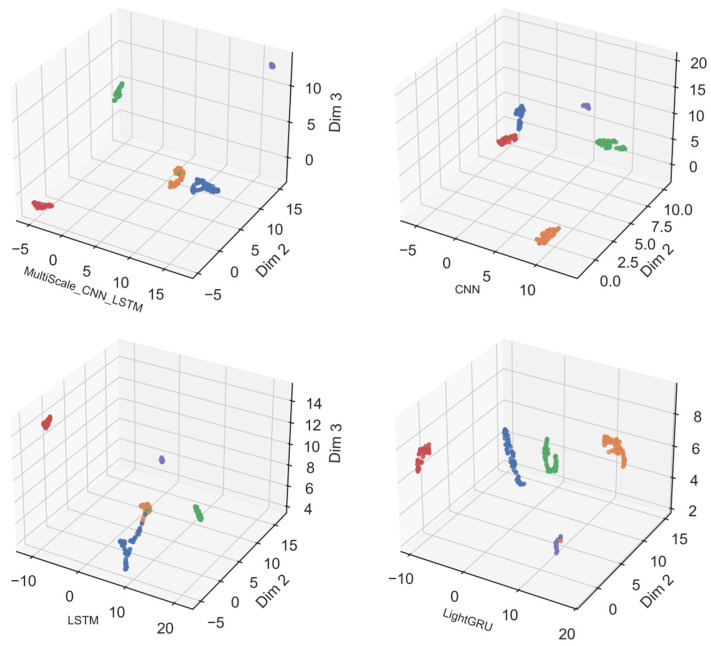
Visualization of feature extraction in three dimensions.

The results in Figure 10 further illustrate the three-dimensional feature distribution of the learned features. This allows us to see the structure of these clusters more clearly. Similar fault types cluster together, demonstrating that the model can effectively identify similar fault patterns. Meanwhile, faults of different categories and normal states remain distinctly separated in the feature space.

Compared with baseline models, the proposed multi-scale CNN-LSTM model exhibits better intra-class compactness and inter-class separation. Features of the same fault type form tighter clusters, and different fault types as well as the normal state are more clearly distinguished. Traditional methods often suffer from overlapping clusters, as they lack the ability to capture subtle fault differences. In contrast, our model achieves more discriminative feature embeddings through refined multi-scale feature extraction.

Visualized experimental results demonstrate the advantages of our method in fault classification, indicating that the model can effectively identify different types of faults and is very suitable for application in short-circuit fault diagnosis in shipboard power systems.

**Figure 11:**
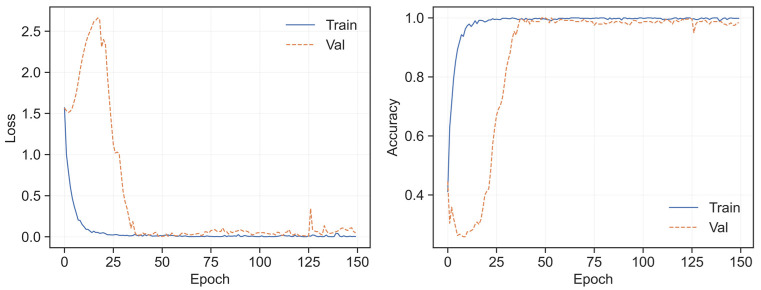
Loss and accuracy of training and validation.

The results in Figure 11 show the training and validation losses drop rapidly at the beginning of training. They eventually stabilize at a low level, without any divergence or rebound. The training and validation accuracies maintain a small gap throughout the training process. They also exhibit highly consistent trends and gradually stabilize.

This stable convergence is attributed to two key design advantages. First, the multi-scale feature extraction module can simultaneously capture local details and global temporal trends of faults, avoiding overfitting caused by relying solely on local features. Second, the temporal modeling ability of the LSTM layer effectively reduces noise interference, ensuring consistent performance between training and validation. The stable training results demonstrate the rationality of the model structure and ensure the reliability of its fault diagnosis results on real ship short-circuit fault datasets.

These experimental results validate the effectiveness of the proposed framework in short-circuit fault diagnosis for shipboard power systems. By combining multi-scale convolutional feature extraction with temporal modeling through LSTM, the framework successfully addresses the challenges posed by varying fault signals. Additionally, the feature-level interpretation and validation step enhances the model’s reliability and interpretability, further supporting the innovations discussed in the introduction.

## 5. Feature Interpretability and Validation

To ensure that the proposed multi-scale CNN-LSTM framework can be reliably applied in shipboard power systems, this study further addresses a more intuitive and crucial question from the “feature level”: why does the model classify a particular short-circuit as a certain type of fault? Which electrical features does it actually rely on? Previously, we have demonstrated through ablation experiments and robustness tests that the model structure design is effective and relatively stable under noise and operating condition fluctuations; however, these experiments mainly indicate whether the model is useful and do not directly explain why the model makes this judgment. Since voltage and current signals in shipboard power systems have clear physical meanings, this section introduces a SHAP-based feature attribution method to quantify and verify the decision basis of the model. The interpretability results are further used for feature selection and input optimization, which supports the claim proposed in the introduction that interpretability-driven model optimization can improve diagnostic accuracy and robustness.

### 5.1. Motivation for Feature-Level Interpretability Analysis

Deep learning models are often referred to as “black boxes.” They can provide classification results, but readers usually cannot see which input information they rely on. For fault diagnosis, this causes two problems. First, in engineering practice, it is difficult to verify whether the model follows real electrical laws. Second, once operating conditions change (such as topology changes, load fluctuations, or increased noise), the model may fail because it relies on unstable or irrelevant features.

Ablation and robustness tests can show whether a module is important and whether the overall system is stable. However, they still cannot directly answer which input features drive the decision. Therefore, this section interprets the model at the feature level and further verifies whether this interpretation is trustworthy. That is, if certain features are important, removing them should significantly degrade performance; conversely, retaining only them should still maintain relatively high performance.

### 5.2. Validation of Feature Importance

**Figure 12:**
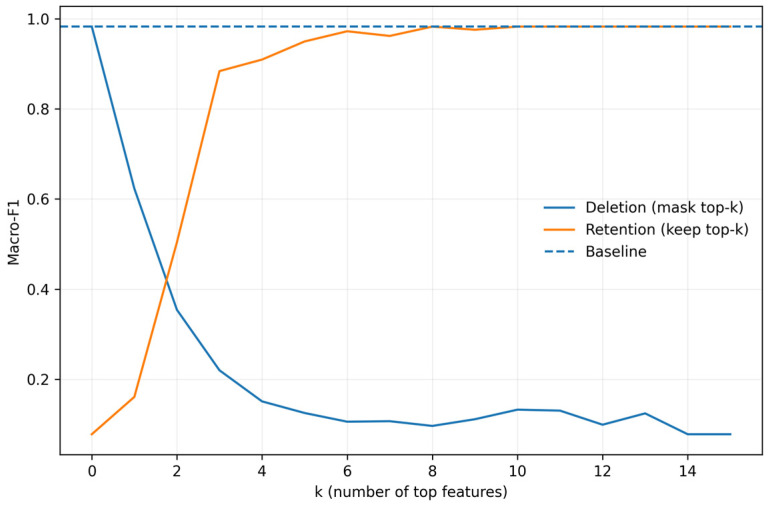
Diagnostic accuracy under feature deletion and retention tests.

Merely providing a feature importance ranking is not sufficient, as “appears important” does not equate to “actually determines performance.” Therefore, we begin with a faithfulness verification: features are ranked from high to low based on SHAP values, followed by two complementary experiments. The first is the retention experiment: only the top-k features are retained, and the model’s accuracy is observed as the number of retained features increases. The second is the deletion experiment: the top-k features are progressively removed, and the resulting changes in accuracy are recorded. The results in Figure 12 show that when only the top-k key features are retained, accuracy increases rapidly as k increases and then stabilizes. This indicates that the model’s primary discriminative information is concentrated in a small number of key features. Conversely, when these key features are gradually deleted, accuracy drops significantly even when only a small number of features are removed. This demonstrates that these features play a decisive role in the model’s decision-making process. In other words, the results in Figure 12 move interpretability from “description” to “verification,” as retention of key features preserves performance, while deletion significantly degrades it. These findings indicate that the importance ranking provided by SHAP is consistent with the model’s performance, providing a reliable basis for optimization based on interpretability analysis.

### 5.3. Physical Interpretation and Model Optimization

**Figure 13:**
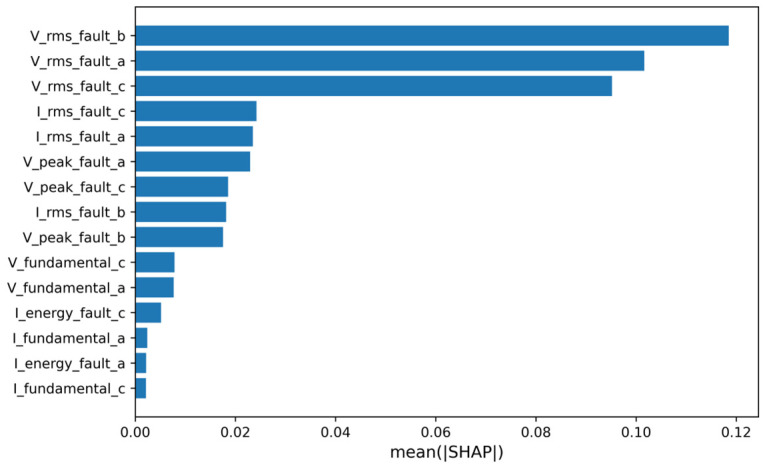
Global feature importance ranking based on mean absolute SHAP values.

This study further clarifies the composition of key features and their physical rationality. The results in Figure 13 present the global feature importance distribution based on the mean absolute SHAP values. Features with high contributions are predominantly concentrated in the post-fault voltage and current amplitude statistics. Among these, phase voltage RMS values (e.g., V_rms_fault_a/b/c) and several current amplitude-related features rank at the top, while fundamental frequency amplitude, energy, and other such features show relatively low contribution. This distribution aligns with the typical electrical response of short-circuit faults: voltage sag and current surge are the most direct and stable fault features, making amplitude features effective for distinguishing fault types and their severity. The similar importance levels of corresponding features across different phases also reflect the structural symmetry and response consistency of the three-phase system under fault conditions.

The global feature ranking from Figure 13 not only serves for model interpretation but also guides the simplification and optimization of input features. Based on the ranking from Figure 13, high-contribution features are prioritized for inclusion in the input set, while low-contribution features are identified for deletion to reduce redundancy or weak relevance. This feature screening strategy is further verified by the results of the retention and deletion experiments in Figure 12: retaining only the top-ranked features allows model performance to quickly stabilize near the baseline, whereas removing these critical features causes a significant drop in performance. Thus, a closed optimization loop is formed: key features are identified and ranked, their causal impacts on model performance are validated, and feature selection and input optimization are conducted accordingly. This process demonstrates that interpretability analysis directly supports feature selection and model optimization, which reduces feature redundancy and noise interference while enhancing the model’s diagnostic accuracy and robustness.

## 6. Conclusions

This study proposes an intelligent fault diagnosis framework based on multi-scale CNN-LSTM for short-circuit faults in shipboard power systems. The framework effectively extracts fault features from non-stationary electrical signals, leading to improved diagnostic accuracy and stable, reliable fault identification. Compared with existing methods, the proposed approach better captures multi-scale transient features and fault dynamic characteristics by exploiting the relative changes between pre-fault and post-fault signals. The Shapley value-based interpretability analysis provides quantitative insights into the contribution of voltage and current features, enabling the optimal selection of input features. The current results indicate that the proposed method maintains relatively stable performance under the tested noise interference conditions. Experimental results demonstrate that the proposed method outperforms traditional fault diagnosis approaches in terms of accuracy, robustness, and interpretability, showing strong potential for engineering applications. Due to the challenges in acquiring real ship fault data and the high risks associated with field tests, the current study uses simulation data for validation and focuses on fault type classification. Future work will focus on collecting real-world data, extending the framework to handle composite short-circuit faults, enabling fault location, and developing real-time interpretability tools to enhance online application capabilities.

## Figures and Tables

**Table 1:** Comparison of fault diagnosis methodologies in the recent literature.

Category	Reference	Core Methodology	Multi-Scale
Traditional ML and Signal Processing	[6,7,8,9,10,11,12,13]	SVM/ANN/DWT/FFT	No
Standard Deep Learning	[14,15,16,17,18,19,20,21]	CNN/LSTM/Transformer/GNN	No
Multi-Scale Deep Learning	[22,23,24,25,26,27]	MS-CNN + BiLSTM/GRU	Yes
Explainable AI (XAI)	[31,32,33,34,35]	XAI-Driven Networks	Varies
Proposed	This paper	Multi-Scale-CNN-LSTM + SHAP	Yes

**Table 2:** Configuration of the proposed multi-scale CNN-LSTM model.

Component	Configuration
Input	Feature sequence
CNN branches	3 parallel Conv1D branches
Kernel sizes	{3, 5, 7}
Filters	32/64
Pooling	MaxPooling1D
Normalization	Batch Normalization
LSTM layers	2
LSTM units	56, 48
Dropout	Applied
Classifier	Fully connected + SoftMax
Optimizer	Adam
Loss function	Cross-entropy

**Table 3:** Performance comparison of different diagnostic models.

Model	Accuracy	Precision	Recall	F1-Score
CNN	99.17 ± 0.34%	99.19 ± 0.34%	99.17 ± 0.34%	99.17 ± 0.34%
LSTM	96.53 ± 0.86%	96.55 ± 0.86%	96.53 ± 0.86%	96.53 ± 0.86%
LightGRU	97.78 ± 0.20%	97.85 ± 0.19%	97.78 ± 0.20%	97.78 ± 0.20%
Multi-Scale CNN-LSTM	99.03 ± 0.20%	99.06 ± 0.19%	99.03 ± 0.20%	99.03 ± 0.19%

## Data Availability

The data are available from the corresponding author on reasonable request.

## References

[1] Latorre A., Soeiro T.B., Geertsma R., Coraddu A., Polinder H. (2023). Shipboard DC Systems—A Critical Overview: Challenges in Primary Distribution, Power-Electronics-Based Protection, and Power Scalability. IEEE Open J. Ind. Electron. Soc..

[2] Fan Z., Liu Y., Guo H., Zhang F., Zeng Y., Wang N. (2026). Overview of fault management for shipboard integrated power system. Electr. Eng..

[3] Latorre A., Soeiro T.B., Fan X., Geertsma R., Popov M., Polinder H. (2024). Pole-to-Pole Short-Circuit Categorization for Protection Strategies in Primary Shipboard DC Systems. IEEE Open J. Ind. Electron. Soc..

[4] Aboelezz A.M., Sedhom B.E., El-Saadawi M.M., Eladl A.A., Siano P. (2023). State-of-the-art review on shipboard microgrids: Architecture, control, management, protection, and future perspectives. Smart Cities.

[5] Binqadhi H., Hamanah W.M., Shafiullah M., Alam S., AlMuhaini M.M., Abido M.A. (2024). A comprehensive survey on advancement and challenges of DC microgrid protection. Sustainability.

[6] Oelhaf J., Kordowich G., Pashaei M., Bergler C., Maier A., Jäger J., Bayer S. (2025). A scoping review of machine learning applications in power system protection and disturbance management. Int. J. Electr. Power Energy Syst..

[7] Vaish R., Dwivedi U.D., Tewari S., Tripathi S.M. (2021). Machine learning applications in power system fault diagnosis: Research advancements and perspectives. Eng. Appl. Artif. Intell..

[8] Aiswarya R., Nair D.S., Rajeev T., Vinod V. (2023). A novel SVM based adaptive scheme for accurate fault identification in microgrid. Electr. Power Syst. Res..

[9] Kurmaiah A., Vaithilingam C. (2025). Optimization of fault identification and location using adaptive neuro-fuzzy inference system and support vector machine for an AC microgrid. IEEE Access.

[10] Bramareswara Rao S.N.V., Kumar Y.V.P., Amir M., Muyeen S.M. (2025). Fault detection and classification in hybrid energy-based multi-area grid-connected microgrid clusters using discrete wavelet transform with deep neural networks. Electr. Eng..

[11] Kuwałek P., Otomański P., Wandachowicz K. (2020). Influence of the phenomenon of spectrum leakage on the evaluation process of metrological properties of power quality analyser. Energies.

[12] Henry M. (2023). An ultra-precise fast Fourier transform. Measurement.

[13] Feng S., Mei Y., Zhang Z., Lei J., Tang Y. (2025). Parameter estimation of power system wideband oscillation based on Zoom FFT and relaxation algorithm. Int. J. Electr. Power Energy Syst..

[14] Zhang L., Zhang Z., Peng H. (2023). Diagnostic Method for Short Circuit Faults at the Generator End of Shipboard power systems Based on MWDN and Deep-Gated RNN-FCN. J. Mar. Sci. Eng..

[15] Thomas J.B., Chaudhari S.G., Verma N.K. (2023). CNN-based transformer model for fault detection in power system networks. IEEE Trans. Instrum. Meas..

[16] Almasoudi F.M. (2023). Enhancing power grid resilience through real-time fault detection and remediation using advanced hybrid machine learning models. Sustainability.

[17] Amiri A.F., Kichou S., Oudira H., Chouder A., Silvestre S. (2024). Fault detection and diagnosis of a photovoltaic system based on deep learning using the combination of a convolutional neural network (cnn) and bidirectional gated recurrent unit (Bi-GRU). Sustainability.

[18] Zhang Y., Chen N., Jiang Y., Jatinkumar V.A. (2023). LSTM-based Fault Prediction for Ship Power Systems. J. Phys. Conf. Ser..

[19] Senemmar S., Zhang J. (2024). Wavelet-based convolutional neural network for non-intrusive load monitoring of next generation shipboard power systems. Meas. Sens..

[20] Senemmar S., Jacob R.A., Zhang J. (2024). Non-intrusive fault detection in shipboard power systems using wavelet graph neural networks. Meas. Energy.

[21] Belagoune S., Bali N., Bakdi A., Baadji B., Atif K. (2021). Deep learning through LSTM classification and regression for intelligent fault identification, diagnosis and location in large-scale multi-machine power system transmission lines. Measurement.

[22] Zhao Z., Li T., Wu J., Sun C., Wang S., Yan R., Chen X. (2020). Deep learning algorithms for rotating machinery intelligent diagnosis: An open source benchmark study. ISA Trans..

[23] Chen X., Zhang B., Gao D. (2021). Bearing fault diagnosis based on Multi-scale CNN and LSTM model. J. Intell. Manuf..

[24] Saghi T., Bustan D., Aphale S.S. (2022). Bearing fault diagnosis based on multi-scale CNN and bidirectional GRU. Vibration.

[25] Liu H., Zhang F., Tan Y., Huang L., Li Y., Huang G., Luo S., Zeng A. (2024). Multi-scale quaternion CNN and BiGRU with cross self-attention feature fusion for fault diagnosis of bearing. Meas. Sci. Technol..

[26] Xu Z., Mei X., Wang X., Yue M., Jin J., Yang Y., Li C. (2022). Fault diagnosis of wind turbine bearing using a multi-scale convolutional neural network with bidirectional long short term memory and weighted majority voting for multi-sensors. Renew. Energy.

[27] Jin Y.R., Qin C.J., Zhang Z.N., Tao J., Liu C. (2022). A multi-scale convolutional neural network for bearing compound fault diagnosis under various noise conditions. Sci. China Technol. Sci..

[28] Hu S., Wu J., Ciren O., Zhu R. (2024). Fault diagnosis of power transformers based on t-SNE and ECOC-TEWSO-SVM. AIP Adv..

[29] Islam M.T., Xing L. (2024). Deciphering the feature representation of deep neural networks for high-performance AI. IEEE Trans. Pattern Anal. Mach. Intell..

[30] Liu Z., Ma R., Zhong Y. (2025). Assessing and improving reliability of neighbor embedding methods: A map-continuity perspective. Nat. Commun..

[31] Aslan E., Ozupak Y., Alpsalaz F., Elbarbary Z.M.S. (2025). A hybrid machine learning approach for predicting power transformer failures using internet of things based monitoring and explainable artificial intelligence. IEEE Access.

[32] Machlev R., Heistrene L., Perl M., Levy K., Belikov J., Mannor S., Levron Y. (2022). Explainable Artificial Intelligence (XAI) techniques for energy and power systems: Review, challenges and opportunities. Energy AI.

[33] Poursaeed A.H., Namdari F. (2025). Explainable AI-driven quantum deep neural network for fault location in DC microgrids. Energies.

[34] Cifci A. (2025). Interpretable prediction of a decentralized smart grid based on machine learning and explainable artificial intelligence. IEEE Access.

[35] Rengasamy P., Rajesh R. (2025). Explainable Artificial Intelligence Framework for Wind Turbine Fault Detection using Random Forest-Extreme Gradient Boosting Hybrid Model. Results Eng..

[36] Cui Y., Wang R., Wang J., Wang Y., Zhang S., Si Y. (2025). Fault diagnosis of ship power grid based on attentional feature fusion and Multi-scale 1D convolution. Electr. Power Syst. Res..

[37] Surucu O., Gadsden S.A., Yawney J. (2023). Condition monitoring using machine learning: A review of theory, applications, and recent advances. Expert Syst. Appl..

[38] Zhao D., Tian C., Fu Z., Zhong Y., Hou J., He W. (2025). Multi scale convolutional neural network combining BiLSTM and attention mechanism for bearing fault diagnosis under multiple working conditions. Sci. Rep..

[39] Chen Y., Zhang R., Gao F. (2024). Fault diagnosis of industrial process using attention mechanism with 3DCNN-LSTM. Chem. Eng. Sci..

